# State capture through indemnification demands? Effects on equity in the global distribution of COVID-19 vaccines

**DOI:** 10.1186/s40545-022-00442-y

**Published:** 2022-08-19

**Authors:** Ariel Gorodensky, Jillian C. Kohler

**Affiliations:** 1 Leslie Dan Faculty of Pharmacy, University of Toronto, Toronto, ON Canada; 2 WHO Collaborating Centre (WHO CC) for Governance, Accountability and Transparency in the Pharmaceutical Sector, Leslie Dan Faculty of Pharmacy, Dalla Lana School of Public Health, University of Toronto, Toronto, ON Canada

**Keywords:** State capture, Corruption, COVID-19, Vaccines, COVAX, Indemnification

## Abstract

**Background:**

State capture by the pharmaceutical industry is a form of corruption whereby pharmaceutical companies shift laws or policies about their products away from the best interest of the public and toward their private benefit. State capture often limits equitable access to pharmaceutical products by inflating drug prices and increasing barriers to entry into the pharmaceutical industry. During the COVID-19 pandemic, the high demand and low supply of COVID-19 vaccines has put governments that manage vaccine procurement at risk of capture by COVID-19 vaccine manufacturers, both through bilateral deals and the COVID-19 Vaccine Global Access (COVAX) Facility; this threatens equity in the global distribution of these products. The purpose of this study is to determine whether COVID-19 vaccine manufacturers have been engaging in state capture and, if so, to examine the implications of state capture on equitable access to COVID-19 vaccines.

**Methods:**

A targeted rapid literature search was conducted on state capture by the pharmaceutical industry. Results were limited to journal articles, books, and grey literature published between 2000 and 2021 in or translated to English. A literature search was also conducted for information about state capture during the COVID-19 pandemic. Results were limited to media articles published between March 2020 and July 2021 in or translated to English. All articles were qualitatively analyzed using thematic analysis.

**Results:**

COVID-19 vaccine manufacturers have demanded financial indemnification from national governments who procure their vaccines. While most high-income countries are legislatively capable of indemnifying vaccine manufacturers, many low- and middle-income countries (LMICs) are not. A number of LMICs have thus changed their legislations to permit for manufacturers’ indemnification demands. Amending legislation in this way is state capture and has led to delays in LMICs and vaccine manufacturers signing procurement contracts. This has critically stalled access to vaccines in LMICs and created disparities in access to vaccines between high-income countries and LMICs.

**Conclusions:**

COVID-19 vaccine manufacturers’ indemnification demands constitute state capture in many LMICs though not in high-income countries; this has enhanced global COVID-19 vaccine inequities. Results underscore the need to find alternatives to financial indemnification that do not hinder critical efforts to end the pandemic.

## Background

### State capture and COVID-19

State capture is the phenomenon by which private enterprises “…bend state laws, policies and regulations to their (mainly financial) benefit through corrupt transactions with public officers and politicians” [1]. It is a form of grand corruption [2] characterized by systemic private influence on the public sphere [3] and which occurs in a “grey zone” of legality [3–5]. Though any private enterprise can engage in state capture, the complexity, high financial stakes, and large number of actors involved in pharmaceutical business result in particularly high capture risks by the pharmaceutical industry [6]. State capture in this instance takes place when pharmaceutical companies shift laws, regulations, or policies about their products away from the best interest of patients and the public, toward the companies’ private benefits [7]. State capture by the pharmaceutical industry limits equitable access to pharmaceutical products by inflating drug prices [8, 9], increasing barriers to entry into the pharmaceutical industry [10], and reducing public trust in healthcare institutions [3]. This undermines universal access to medicines and other pharmaceutical products, which is crucial to achieving Universal Health Coverage (UHC) and in promoting health as a human right [11].

Governments are most likely to be captured when power is concentrated in one or a few large private enterprises as these companies control—and hold tremendous bargaining power over—their resources [12]. During the COVID-19 pandemic, a few large transnational pharmaceutical companies have held near-monopolies over personal protective equipment (PPE), COVID-19 therapeutics and diagnostics, and most recently, vaccines [13]. Governments’ urgent needs for these products, combined with the highly concentrated global health product and pharmaceutical market, have resulted in tremendous power imbalances between governments who are in desperate need of COVID-19 vaccines, and manufacturers who produce these vaccines [13]. This puts national governments at risk of capture by the pharmaceutical industry [14], and, as a result, threatens to hinder equity in the global distribution of COVID-19-related products.

The COVID-19 pandemic has demonstrated that health systems around the world share vulnerabilities [15]; that is, as Director General of the World Health Organization (WHO) Dr. Tedros Adhanom Ghebreyesus has asserted, “…no one is safe until we are all safe” from this virus [16]. Addressing these vulnerabilities requires non-discriminatory, universal, and equitable access to COVID-related healthcare [11]. Specifically, ending the acute phase of the pandemic urgently requires the global equitable distribution of COVID-19 vaccines [17]. As such, vaccine equity has become an urgent moral, strategic, and economic imperative [18].

Current COVID-19 vaccine distributions are guided by bilateral agreements between national governments and pharmaceutical companies as well as by COVID-19 Vaccine Global Access (COVAX), a multilateral initiative intended to facilitate the global equitable distribution of COVID-19 vaccines [19, 20].

COVAX was established in April of 2020 as part of the Access to COVID-19 Tools (ACT) Accelerator: an effort to facilitate the global equitable distribution of COVID-19 tools [21]. The ACT Accelerator has 4 pillars, each of which supports the distribution of one type of tool: COVID-19 therapeutics, diagnostics, vaccines, or other emergency health system support [22].

COVAX, the Accelerator’s vaccine pillar, was established with the purpose of ending the acute phase of the pandemic by the end of 2021 by facilitating the global equitable distribution of COVID-19 vaccines [23]. COVAX aims to do this by first “…pool[ing] resources and shar[ing] vaccine development risk” to diversify vaccine portfolios, then by facilitating the deployment of safe, effective, and affordable COVID-19 vaccines around the world via the COVAX Facility [20] (All countries–UN member states and non-UN member states–can opt into the COVAX Facility [19]). In so doing, COVAX’s initial goal was to procure 2 billion vaccine doses by the end of 2021 [19].

While the COVAX Facility has potential to contribute to global equitable vaccine deployment, to date, it has missed its mark. According to UNICEF (2022), COVAX delivered only 910.4 million vaccine doses in 2021, falling short of its initial 2 billion target by more than 1 billion shots [24]. What’s more, COVAX has largely failed to reduce global disparities in access to COVID-19 vaccines; bilateral deals between high-income countries and vaccine manufacturers have limited the global supply of COVID-19 vaccines available for purchase by the COVAX Facility [25]. As such, the Facility has experienced vaccine shortages that have resulted in cuts to vaccine distributions and forecasts [25]. Today, only 17.5% of the vaccine doses administered around the world have been delivered through the COVAX Facility; as a result, while high-income countries have purchased enough vaccines to fully vaccinate their populations three times over, only 8.5% of individuals in low-income countries have received at least one dose [26, 27]. It seems, therefore, that COVAX is more of a way for high-income countries and vaccine manufacturers to deflect their obligations to support global and public health than it is for low-income countries to access life-saving vaccines.

### Financial indemnification of vaccine manufacturers during public health emergencies

Typically, when pharmaceutical companies develop a novel product, they secure insurance policies that cover any financial compensation required from legal action in the case of severe adverse events (SAEs) caused by their product [28]. During public health crises, however, the speed with which products are developed and rolled-out means that manufacturers forego typical insurance processes [28, 29]. Instead, to ensure they are protected against financial liability for SAEs, manufacturers include product indemnification clauses^1^ within procurement contracts [28]. These are clauses that outline the responsibility of national governments (the indemnifying party) to cover financial losses incurred by COVID-19 vaccine manufacturers (the indemnified party) as a result of SAE lawsuits [30]. Once governments sign these contracts, they assume financial liability for SAEs caused by manufacturers’ products [31]. In short, financial accountability for adverse events caused by pharmaceutical manufacturers’ products is transferred from manufacturers to states.

During the 2009 H1N1 pandemic, as an example, H1N1 vaccine manufacturers demanded indemnification for financial liability from governments who procured their vaccines [32]. Now, during the COVID-19 pandemic, many COVID-19 vaccine manufacturers are demanding full financial indemnification from countries that receive their vaccines via bilateral negotiations with COVID-19 vaccine manufacturers and through the COVAX Facility [32–34].

In order for a country to indemnify a vaccine producer, its national legislation must permit for the full financial indemnification of a goods manufacturer [35, 36]. In countries without this legislation, signing COVID-19 vaccine procurement contracts requires amending or creating new national laws. When the revision or addition of legislation occurs as a result of private companies’ influence on the public sphere [3], is catalyzed by power imbalances between companies and governments [12], is not necessarily illegal [3–5], and results in laws, policies, or regulations that benefit one or many private enterprises [7], it is state capture.

### Rationale and aim

As a result of the rapidly evolving state of the pandemic, the speed with which COVID-19 vaccines are being rolled-out, and general secrecy about corruption, there is a dearth of scholarly information about state capture in the global distribution of COVID-19 vaccines. Given that state capture can impact equitable access to pharmaceutical products and the urgent need for COVID-19 vaccine equity, it is important to examine whether COVID-19 vaccine manufacturers’ indemnification demands constitute state capture and to explore the impact these demands have on global equitable access to COVID-19 vaccines; an examination of state capture in global COVID-19 vaccine procurements is thus warranted.

In this paper, we will argue that COVID-19 vaccine manufacturers’ indemnification demands can constitute state capture. Specifically, we will demonstrate that indemnification clauses within bilateral vaccine supply agreements and through the COVAX Facility amount to state capture in many low- and middle-income countries (LMICs), though not in many high-income countries (HICs); we will also explore the implications that this state capture has on the global equitable distribution of COVID-19 vaccines.

## Methods

### Search strategy

A targeted rapid literature search was conducted in two parts to collect general information about state capture by the pharmaceutical industry, then to explore state capture by COVID-19 vaccine manufacturers during the COVID-19 pandemic more specifically. First, literature searches were conducted about state capture by the pharmaceutical industry generally. These searches were conducted between February and March of 2021 in seven databases (SCOPUS, PubMed, Google Scholar, JSTOR, ProQuest, SSRN Pharmaceutical Science Research Network, and Sage Journals). Inclusion criteria were: (1) peer-reviewed journal articles, books, and grey literature; (2) published between 2000 and 2021; (3) published in or translated to English; and (4) contained information about how the pharmaceutical industry engages in state capture, what are the impacts of state capture by the pharmaceutical industry on the healthcare sector, and/or about how public institutions can mitigate their risks of capture by pharmaceutical companies. There were no exclusion criteria for these searches.

Next, a series of literature searches were conducted in the ProQuest database for media articles related to state capture in the global distribution of COVID-19 vaccines; the searches were conducted in July of 2021. Inclusion criterial were: (1) media articles and wire feeds; (2) written in or translated to English; (3) published between March 2020 and July 2021; and (4) contained information about state capture by the pharmaceutical industry during the COVID-19 pandemic. Results were limited to media articles and wire feeds as these source types provided timely yet reliable information about state capture in the global distribution of COVID-19 vaccines. There were no exclusion criteria for this search. Articles from both sets of literature searches were assessed for relevance through (1) title and abstract screens; and (2) full text reviews.

### Data extraction and analysis

Following screening, data from 270 articles were extracted and analyzed via thematic analysis. Articles were qualitatively analyzed according to their relationship to themes such as “state capture in bilateral deals”, and “state capture through COVAX”. Furthermore, literature was analyzed for its general relevance to state capture by COVID-19 vaccine manufacturers during the COVID-19 pandemic. The results section of this paper is presented according to the themes identified during data analysis.

## Results

### State capture through bilateral agreements

Most COVID-19 vaccine manufacturers (i.e., Pfizer-BioNTech, Moderna, Serum Institute of India) have demanded financial indemnification from countries who procure their products so that the companies can minimize their financial risks and maximize their profits [37–39]. Given that governments have little bargaining power when in a public health crisis, they are inclined to sign indemnification agreements in order to rapidly procure the emergency vaccines they need [40]. High-income countries (HICs) are often already legislatively capable of signing indemnification agreements with vaccine manufacturers [31, 41]. As such, signing indemnification agreements in many high-income countries does not necessitate making amendments to existing legislation or creating new national laws, and thus, does not constitute state capture.

In the United States (U.S.), for instance, the 2005 *Public Readiness and Emergency Preparedness Act* “…provides immunity from liability…” to vaccine manufacturers during public health emergencies [41, 42]. As a result, following the Trump Administration’s declaration of COVID-19 as a public health emergency in February of 2020, the United States government was already legislatively capable of signing indemnification agreements with COVID-19 vaccine producers [42]. Similarly, the *Canadian Pandemic Influenza Preparedness: Planning Guidance for the Health Sector* states that “to prevent delays in release of the vaccine at time of pandemic…the Government of Canada will indemnify the manufacturer against any claims or lawsuits brought against it by third parties” [43]. During an influenza pandemic, therefore, the Canadian government is legislatively capable of indemnifying vaccine manufacturers.

Furthermore, many HICs are willing and able to accept the financial risks of indemnifying vaccine producers. They often have the financial means necessary to cover the costs of indemnification and are often willing to risk public funds in order to access life-saving products. In Great Britain, for instance, when COVID-19 vaccine procurement contracts became available, there was a general consensus that “The public purse may incur additional costs…” as a result of indemnification agreements [44]. The United Kingdom government was nonetheless willing and able to incur these costs and indemnify COVID-19 vaccine manufacturers.

Although many HICs are capable of indemnifying vaccine manufacturers, “…there is far less room for compromise for poorer nations” [45]. Many LMICs are not legislatively capable of indemnifying vaccine manufacturers. As such, signing indemnification agreements would require these governments to amend existing—or create new—national laws [36, 46]. In Colombia, for instance, at the request of a number of pharmaceutical companies, the government passed new legislation that permitted the indemnification of COVID-19 vaccine manufacturers [47]. The contract between the Colombian government and Pfizer-BioNTech, leaked in the Colombian media, thus states that the

Purchaser [Colombia] hereby agrees to indemnify, defend and hold harmless Pfizer, BioNTech, each of their Affiliates…from and against any and all suits, claims, actions, demands, losses, damages, liabilities, settlements, penalties, fines, costs and expenses…caused by, arising out of, relating to, or resulting from the Vaccine, including but not limited to any stage of design, development, investigation, formulation, testing, clinical testing, manufacture, labeling, packaging, transport, storage, distribution, marketing, promotion, sale, purchase, licensing, donation, dispensing, prescribing, administration, provision, or use of the Vaccine, any information, instructions, advice or guidance provided by Pfizer or BioNTech or any of their respective Affiliates and relating to the use of the Vaccine, or any processing or transfer of anyone’s personal information processed and transferred by Purchaser to the Indemnitees…

Additionally, at date of writing, 13 Latin American countries have amended existing or implemented new legislation in order to reach indemnification agreements with COVID-19 vaccine manufacturers [36, 46]. Many Latin American countries including Peru and Argentina have also modified existing confidentiality laws to meet vaccine manufacturers’ stringent confidentiality demands that have prohibited them from publishing information such as price per vaccine dose [46].

This need to amend or create new legislation has occurred as a result of private pharmaceutical companies’ influence on the public sphere and is catalyzed by power imbalances between national governments and COVID-19 vaccine manufacturers (wherein the high demand for—and low supply of—COVID-19 vaccines puts pharmaceutical companies in positions of strength [13]). Though this is technically legal, it results in national laws that financially benefit one or many private enterprises; this is state capture.

In Argentina, as an example, legislation has not historically permitted the financial indemnification of vaccine manufacturers [48]. In their early negotiations with the country, however, Pfizer-BioNTech (hereafter referred to as Pfizer) demanded that Argentina indemnify the company against SAE lawsuits [35, 49]. As a result, in November of 2020, Argentine officials passed Law 27.573 (referred to as the *Vaccine Law*), implemented to regulate the public purchase of emergency vaccines including those for COVID-19 [35, 49]. Article 4 of the Law states that the Argentine government may sign “clauses establishing conditions of indemnity of property in respect of compensation and other pecuniary claims…with the exception of those originating in fraudulent maneuvers, malicious conduct or negligence” [35].

Argentine officials allegedly “tailored” the *Vaccine Law* to Pfizer’s requests [50]. The creation of this law thus resulted from Pfizer’s influence on Argentine authorities and led to the creation of national legislation that benefitted Pfizer, suggesting that Pfizer captured Argentina.

#### Implications for equity in the global distribution of COVID-19 vaccines

State capture via vaccine manufacturers’ indemnification demands have led to delays in reaching bilateral COVID-19 vaccine procurement agreements. This has occurred because amending or creating new national legislation is a lengthy, complicated, and costly process. Since delays are not experienced in HICs where national legislation already permits the full indemnification of vaccine manufacturers, high-income countries are able to receive COVID-19 vaccines far more rapidly than LMICs; that is, once LMICs are legally able to sign vaccine procurement agreements with manufacturers, vaccine availability is limited because it excludes doses already earmarked for HICs. This hinders equity in the global distribution of COVID-19 vaccines and amplifies health-related and socioeconomic disparities between HICs and LMICs.

In Colombia, for example, vaccine manufacturers’ indemnification demands required that the country pass a new law permitting for the financial indemnification of manufacturers [47]. The process of creating this law led to delays in the country signing procurement contracts with vaccine manufacturers, and thus, in receiving COVID-19 vaccines [47]. Similarly, in an unnamed Latin American country, Pfizer’s demands for SAE indemnification led to a 3-month delay in reaching an agreement with the national government, which delayed the country’s receipt of the vaccines [51].

Even after Argentina’s enactment of the *Vaccine Law*, Pfizer refused to sign a vaccine procurement agreement with the nation; this was because they were dissatisfied with Argentina’s conditions for indemnification. Pfizer did not agree with the clause that the country would not indemnify them against SAEs “…originating in fraudulent maneuvers, malicious conduct or negligence” [35]. Specifically, Pfizer demanded that Argentine officials remove the word “negligence” from the exceptionalities in their law, as its inclusion would open them up to a wider level of responsibility than they were willing to accept [13]. Argentine officials, however, refused to make the amendment, as they were not willing to accept Pfizer's demands for higher-level non-liability, including that Argentina would “…take care of whether a batch came in poor condition, broke the cold chain or [was]…diluted with water” [52]. As a result, Argentina was not able to sign an agreement with the manufacturer, and thus, was not able to receive their vaccines [53].

Reports suggest that Pfizer’s demands in Argentina were likely a “strategy” to ensure that the company would not commit to a contract with the country [53, 54]. Former Argentine Minister of Health Ginés González García speculated that vaccine shortages had resulted in Pfizer struggling to fulfill their bilateral contracts in other (mostly high-income) countries and that a contract with Argentina would exacerbate shortages and prolong delays in vaccine shipments to these countries [53, 54]. This illuminates the inequities in the global distribution of COVID-19 vaccines and demonstrates how indemnification demands can hinder access to vaccines in LMICs, thus exacerbating vaccine disparities between HICs and LMICs.

In addition, countries unable to receive vaccines via bilateral agreements can access COVID-19 vaccines through bilateral donations from other countries. In order for a country to receive donated vaccines, however, the recipient country must already have an indemnification agreement in place with the company whose vaccines will be donated [55]. As a result, if a country is unable, for any reason, to reach an indemnification agreement with a COVID-19 vaccine manufacturer, they will not be able to receive that manufacturer’s vaccine via donations from other countries.

In June of 2021, for instance, the United States planned to donate 500 million doses of Pfizer’s COVID-19 vaccine to a number of countries including Argentina [55]. Since, however, Argentina was unable to reach a bilateral agreement with Pfizer, the country was also unable to receive donated vaccines from the United States [55]. This demonstrates that COVID-19 vaccine manufacturers’ indemnification demands can hinder access to vaccines by inhibiting not only the speed at which LMICs can procure vaccines through bilateral agreements, but by eliminating the availability of vaccine donations from other countries as well.

### State capture through the COVAX Facility

For countries that choose not to or cannot procure vaccines through bilateral procurement deals with manufacturers, the only remaining mechanism by which they can receive internationally produced COVID-19 vaccines is through the COVAX Facility. The COVAX Facility, however, has been experiencing vaccine supply shortages, thus rendering vaccine access for those who rely on the Facility particularly challenging [56].

Additionally, COVAX requires that all participants who receive vaccines through the COVAX Facility indemnify “…manufacturers, donors, distributors, and other stakeholders…against any losses they incur from the deployment and use of…” COVID-19 vaccines [32]. The Facility operates under the assumption that indemnifying vaccine manufacturers is necessary, as manufacturers will choose not to send vaccines to countries that hold them financially liable for SAEs [28]. In recognition that indemnification may be financially challenging for AMC participants, COVAX has established a no-fault, lump-sum compensation program, through which any individual who receives a COVID-19 vaccine in an AMC country and experiences an SAE can receive financial compensation [32]. If a claimant accepts compensation through this program for an adverse event suffered as a result of a COVID-19 vaccine, however, they waive their right to litigate against the vaccine manufacturer for damages as a result of that event; thus, COVAX’s no-fault compensation program offers a solution to governments’ financial challenges with indemnifying vaccine manufacturers for SAEs caused by COVID-19 vaccines, though does not address legal hurdles to indemnifying manufacturers in the first place [32].

The COVAX Facility is the only mechanism that countries without bilateral agreements with vaccine manufacturers can turn to in order to receive internationally produced COVID-19 vaccines. Countries without the legal capacity to indemnify vaccine manufacturers who wish to receive vaccines through the Facility must amend existing or create new national laws. In the Philippines, for example, president Rodrigo Duterte modified national legislation to permit for the creation of a COVID-19 vaccine indemnification fund, with the purpose of receiving vaccines through the COVAX Facility [57]. Legislative changes such as this one are catalyzed by power imbalances between national governments that have no other mechanism of receiving internationally produced vaccines and COVID-19 vaccine manufacturers and they result in national laws that financially benefit one or many private enterprises; thus, these changes constitute state capture.

Pfizer is allegedly using the same procurement contracts with national governments procuring their vaccines through bilateral agreements as with those procuring their vaccines via the COVAX Facility [58]. In its contract with Argentina, for example, COVAX demanded that officials change the *Vaccine Law* to permit for indemnification in cases of negligence [58]. As in its bilateral negotiations, Argentine officials did not accede to this [59, 60]. Given that COVAX was their last opportunity to receive Pfizer’s vaccines, Argentina was left entirely without access to Pfizer’s product [59, 60].

While Argentina has received other manufacturers’ vaccines (such as Russia’s Sputnik-V and AstraZeneca’s COVID-19 vaccine), Pfizer’s is one of only a few (others include the Moderna and Sinopharm COVID-19 vaccines) to be approved in Argentina for children under the age of 18 [61, 62]. The City Health Minister for Buenos Aires, Fernán Quirós, stated in July of 2021 that “…having a legal instrument that allows access to vaccines that we are not able to access today seems to be extremely relevant, especially vaccines that are approved for adolescents…” [63]. On July 2, 2021, therefore, Argentine President Alberto Fernández amended Article 4 of the *Vaccine Law* to meet Pfizer’s demand to remove negligence from the law’s exceptionalities [64]. The modified *Vaccine Law* permits “…clauses that establish conditions of patrimonial indemnity regarding indemnities and other pecuniary claims related to and in favor of those who participate in research, development, manufacture, supply and supply of vaccines, with the exception of those originated in malicious conduct on the part of the aforementioned subjects” [65]. This amendment constitutes state capture. As a result of Pfizer’s power over Argentina and their sustained pressure to amend existing legislation, national officials changed the *Vaccine Law*—to Pfizer’s benefit—in order to receive a product that they desperately needed. On July 27, 2021, Argentina and Pfizer signed their first deal: a contract for 20 million doses of the Pfizer-BioNTech vaccine, to be delivered before the end of the year [66].

#### Implications for equity in the global distribution of COVID-19 vaccines

For many low-resource countries that do not have the financial ability or bargaining power necessary to procure vaccines through bilateral deals with manufacturers, COVAX is the only mechanism through which they can receive internationally produced COVID-19 vaccines. The obligation that countries indemnify all COVID-19 vaccine manufacturers thus leaves many LMICs with only two options: amend their legislation to permit for the indemnification of vaccine manufacturers or forego receiving COVID-19 vaccines altogether. Indemnification demands within the COVAX Facility’s procurement contracts, therefore, can have serious implications for access to vaccines.

In June 2021, for example, Argentina had missed out on approximately 8 million doses of Pfizer’s COVID-19 vaccine as a result of the company’s indemnification demands [67]. Given that the COVAX Facility obliges participating countries to indemnify vaccine manufacturers, they were not able to assist Argentina in receiving the emergency vaccines they needed. According to the former Argentine Minister of Health, Ginés González García, therefore, “…COVAX did not work…it did not fulfill what was its purpose [to facilitate the global equitable distribution of COVID-19 vaccines]” [49].

## Discussion

This paper is a preliminary examination of how state capture manifests in COVID-19 vaccine manufacturers’ indemnification requirements and the implications that state capture has had for equity in the global distribution of COVID-19 vaccines. Vaccine inequities may result in HIC vaccinations being complete years before LMIC ones, so there is growing concern that vaccine inequities will prolong the acute phase of the COVID-19 pandemic [68]. This study thus confirms existing accounts of vaccine inequities and their serious implications for efforts to end the COVID-19 pandemic [11, 26, 27, 69].

According to study results, the procurement of COVID-19 vaccines has mostly occurred under “…a cloak of opacity” [36]. In addition to indemnification demands, most COVID-19 vaccine manufacturers have included confidentiality agreements within their vaccine contracts [36, 46, 70]. As a result, little public information is available about contract terms, including details in most indemnification clauses, prices per dose, and delivery schedules [46, 71, 72]. National governments are thus unable to compare contract terms with other countries, so cannot engage in informed negotiations [46]. This lack of transparency amplifies power imbalances between vaccine producers and governments, which limits accountability for the deployment of safe and effective vaccines. Contract secrecy also reduces countries’ bargaining power, which hinders access to affordable vaccines, so further inhibits equity in vaccine distribution.

This study confirms existing literature describing the importance of transparency in promoting accountability [73–75] and delineates the impacts of contract opacity on equitable COVID-19 vaccine distribution [30]. Rhodes et al. explain, for example, that COVID-19 vaccine procurement contract opacity has resulted in differential COVID-19 vaccine pricing inversely proportional to countries’ gross domestic products [30]. The higher price per vaccine dose that LMICs are paying compared to their HIC counterparts has hindered global equitable access to COVID-19 vaccines.

While indemnification demands have become commonplace during public health emergencies, the implications that these demands have for global equitable access to life-saving vaccines calls into question their necessity. Vaccine manufacturers, for example, typically cite their large research and development expenditures and their rapid product rollout as necessitating indemnification agreements so that they can profit from their products [51, 76]. During the COVID-19 pandemic, however, in the interest of accelerating the development of COVID-19 vaccines, a number of countries subsidized manufacturers’ R&D processes [77–79]. As a result, according to Clarín David Larios, President of the Spanish Lawyers for Health Associations, indemnification “…is an excessive clause. [Manufacturers] are already being given public money for vaccine research and development, they are paid for vaccines and if we also have to pay for adverse effects, we are paying three times” [77]. Halabi, Heinrich, and Saad (2020) have also explained that some governments refuse indemnity agreements on the basis of “…fairness principles: manufacturers should pay for the injuries their products cause [80].” This study thus supports existing literature describing common oppositions to indemnities for severe adverse events during public health emergencies and adds to this literature by providing evidence of the implications of indemnification demands on the global equitable distribution of COVID-19 vaccines.

Furthermore, to demonstrate that financial indemnification is not necessary for the successful rollout of an emergency health product, Russia’s Sputnik-V COVID-19 vaccine producers have not demanded indemnification from recipient countries [37]. According to Russian officials, “We are putting our money where our mouth is by not asking for full indemnity in partnerships we create in different countries” [37]. This demonstrates that, while indemnification demands are the norm during public health emergencies, they are certainly not necessary; emergency health products can rapidly and efficiently be rolled-out without indemnification agreements.

There are two notable limitations to this study. First, results were limited to publicly available information. Given that there are confidentiality clauses in most COVID-19 vaccine procurement contracts, it is likely that there is information about vaccine manufacturers’ indemnification demands that is not publicly available, and thus, which could not be included in this investigation. Secondly, data about manufacturers’ indemnification demands during the COVID-19 pandemic were collected from media articles and wire feeds only, and thus, are not peer-reviewed. These articles, however, provide information that is not available elsewhere. As a result, this study highlights the importance of the media in times of crisis and the crucial role the media plays in publicizing corruption during public health emergencies.

## Conclusions

Results indicate that COVID-19 vaccine manufacturers’ indemnification demands, both within bilateral agreements and through the COVAX Facility, constitute state capture in many LMICs though not in many HICs. These demands limit LMICs’ abilities to sign vaccine procurement contracts with COVID-19 vaccine manufacturers, and thus, their ability to receive vaccines through bilateral negotiations and through donations from other countries. Similarly, the COVAX Facility’s policy that all participating countries must indemnify vaccine manufacturers can hinder governments from signing COVAX procurement contracts; this limits the COVAX Facility’s ability to achieve its intended purpose of facilitating the global equitable distribution of COVID-19 vaccines. These results underscore the need to reduce power imbalances between governments and pharmaceutical companies and to find alternatives to indemnification that do not impede the equitable distribution of emergency vaccines or efforts to end the COVID-19 pandemic.

## Data Availability

All documents used for this research are publicly available.

## Abbreviations

COVAX: COVID-19 Vaccine Global Access
LMICs: Low- and middle-income countries
PPE: Personal protective equipment
WHO: World Health Organization
ACT accelerator: Access to COVID-19 Tools accelerator
SAE: Severe adverse event
HIC: High-income country
U.S.: United States
R&D: Research and Development

## Footnotes

1 It is important to note that financial indemnities are distinct from no-fault vaccine injury compensation schemes. For government indemnification to occur, a patient must successfully litigate against a vaccine manufacturer [32, 80]. To reap compensation for vaccine-related injuries through no-fault compensation schemes, however, patients do not need to prove fault; in some jurisdictions such as the United States, patients who make a claim to a compensation scheme waive their right to bring a civil case against the vaccine manufacturer [32, 80, 81].
